# Alteration in the Cytokine Secretion of Bone Marrow Stromal Cells from Patients with Chronic Myelomonocytic Leukemia Contribute to Impaired Hematopoietic Supportive Activity

**DOI:** 10.1155/2018/5921392

**Published:** 2018-07-10

**Authors:** Hui Shi, Yingshao Wang, Rong Li, Wen Xing, Feng-Chun Yang, Jie Bai, Yuan Zhou

**Affiliations:** ^1^State Key Laboratory of Experimental Hematology, Institute of Hematology & Blood Diseases Hospital, Center for Stem Cell Medicine, Chinese Academy of Medical Sciences & Peking Union Medical College, Tianjin, China; ^2^Department of Biochemistry and Molecular Biology, University of Miami Leonard M. Miller School of Medicine, Miami, FL, USA; ^3^Sylvester Comprehensive Cancer Center, University of Miami Leonard M. Miller School of Medicine, Miami, FL, USA; ^4^Department of Hematology, The Second Hospital of Tianjin Medical University, Tianjin, China

## Abstract

Bone marrow stromal cells (BMSCs) represent an important cellular component of the bone marrow microenvironment, which play an important role in supporting and regulating the proliferation and differentiation of hematopoietic stem/progenitor cells (HSPCs). We have previously reported that the ability of BMSCs derived from CMML patients (CMML-BMSCs) in supporting the expansion of cord blood (CB) CD34^+^ cells was significantly reduced compared to BMSCs derived from healthy donors (HD-BMSCs). In addition, CMML-BMSCs led to a skewed differentiation of CB CD34^+^ cells favoring myeloid lineage compared with HD-BMSCs. To assess whether the altered cytokine secretion was one of the mechanisms to mediate the impaired hematopoietic supportive activity of CMML-BMSCs, a transwell coculture followed by cytokine array was performed. We showed that noncontacted coculture with CMML-BMSCs preferentially promoted the differentiation of CB CD34^+^ cells toward myeloid lineage. The expression levels of multiple cytokines (IL-6, IL-8, and GRO-*β*) were markedly reduced in CMML-BMSCs compared with HD-BMSCs. By supplementing IL-6, IL-8, or GRO-*β*, the hematopoietic supportive activity of CMML-BMSCs was partially restored. These results suggested that BMSCs may contribute to the pathogenesis of CMML by altering their cytokine secretion, which will shed light on the further investigation to develop novel therapeutic strategies for CMML patients.

## 1. Introduction

Bone marrow (BM) microenvironment plays an important role in regulating cell fates and lineage commitment of hematopoietic stem/progenitor cell (HSPCs) [1–3]. Bone marrow stromal cells (BMSCs) are the major component of the BM microenvironment [4–8]. Several experimental and clinical findings suggest that the abnormal biological functions of BMSCs might contribute to the initiation and progression of hematopoietic disorders. For example, the genetic alteration of the mesenchymal/osteoblastic lineage can induce myelodysplastic syndrome- (MDS-) like phenotypes in mice which supports the notion that niche can be the key factor for the pathogenesis of hematopoietic malignancies [9]. Studies by Walkley et al. using *RARγ^−/−^* mice and *Rb-null* mice show that in addition to the intrinsic defects of hematopoietic cells, defective microenvironment played an important role in the initiation and progression of myeloproliferative syndromes [10, 11]. Furthermore, other studies show that defects in the BMSCs can occur during aging and may have a direct impact in the development of hematological disorders such as MDS [12–14], multiple myeloma [15], and acute myeloid leukemia (AML) [16, 17].

Chronic myelomonocytic leukemia (CMML) is a clonal disorder of hematopoietic stem cells (HSCs) that is characterized by the presence of peripheral blood (PB) monocytosis with overlapping features between MDS and myeloproliferative neoplasm (MPN) and a risk of transformation to AML [18, 19]. There has been no tremendous improvement in the therapeutic strategies for this disease for several decades, and the therapeutic effects for patients with CMML remain unsatisfactory. We recently observed that human umbilical cord blood (CB) CD34^+^ cells cocultured with BMSCs derived from CMML patients (CMML-BMSCs) had impaired colony-forming capacity with a myeloid differentiation bias, compared to those cocultured with BMSCs derived from healthy donors (HD-BMSCs) [20]. In the current study, we compared the hematopoietic supportive activity of CMML-BMSCs with HD-BMSCs in a transwell system. We showed that CMML-BMSCs exhibited impaired hematopoietic supportive activity and promoted CB CD34^+^ cell differentiation toward myeloid cells without direct cell-cell contact. Furthermore, we observed that multiple cytokine secretions were significantly decreased in CMML-BMSCs compared with HD-BMSCs, which may result in the reduction in hematopoietic supportive activity. In addition, adding the decreased cytokines back to the transwell coculture system partially restored the hematopoietic supportive activity of CMML-BMSCs as evidenced by increased numbers of total colony-forming unit cell (CFU-C) and colony-forming unit granulocyte, erythrocyte, monocyte, and megakaryocyte (CFU-GEMM). These results provide evidence that BMSCs may contribute to the pathogenesis of CMML through altered cytokine secretion, which will help us to develop novel therapeutic strategies for CMML patients who are mostly treated on palliative drugs and supportive care.

## 2. Materials and Methods

### 2.1. Patients

Thirteen patients with CMML (10 males and 3 females) and 10 healthy donors (7 males and 3 females) were included in this study. The study was approved by the Ethics Committee of the Institute of Hematology and Blood Diseases Hospital, Chinese Academy of Medical Sciences, according to guidelines of the 1975 Helsinki Declaration, and informed consent was received according to the institute's guidelines on the use of human subjects. All patients were reevaluated and met the 2008 WHO diagnostic criteria.

### 2.2. Isolation and Expansion of BMSCs

Whole BM cells from CMML patients and healthy donors were cultured at 4 × 10^6^ cells/well in a 6-well plate at 37°C, 5% CO_2_, 5% O_2_ in a fully humidified atmosphere in Dulbecco's modified Eagle's medium (DMEM)/F12 (Gibco, Carlsbad, USA) containing 10% fetal bovine serum (FBS, HyClone, South Logan, USA), 1x insulin-transferrin-selenium A (Life Technologies, Carlsbad, USA), 10 ng/mL human epidermal growth factor (EGF, PeproTech, Rocky Hill NJ, USA), and 10 ng/mL human platelet-derived growth factor-BB (PDGF-BB, PeproTech, Rocky Hill NJ, USA) (expansion medium). Photographs were taken by a Fujifilm digital camera (FinePix 2400 Zoom, Fujifilm, Tokyo, Japan). For functional analysis, cells were trypsinized and replated after reaching 80% confluence and BMSCs at passages 3–5 were used for the following experiments.

### 2.3. Phenotypic Analysis of BMSCs

The phenotypic analyses of BMSCs were performed by evaluating the expression of surface markers on a FACS LSR II flow cytometer (Becton Dickinson, San Jose, USA). In brief, BMSCs were incubated with CD45, CD34, CD31, CD73, CD105, CD44, CD29, and CD90 antibodies (BD Pharmingen, San Diego, CA) alone or in combination for 30 min at 4°C, then washed with PBS containing 0.1% bovine serum albumin and analyzed by flow cytometry.

### 2.4. Transwell Coculture Assay

Transwell coculture assay was performed to determine the effects of BMSCs on CB CD34^+^ cells without direct cell-cell contact. In brief, mononuclear cells from CB were separated by Ficoll-Hypaque (Sigma, Munich, Germany) density gradient centrifugation. CB CD34^+^ cells were purified by using magnetic microbeads following the manufacturer's instructions (Miltenyi, Bergisch Gladbach, Germany). CB CD34^+^ cells (2 × 10^4^) were then placed in the transwell insert (Costar Transwell® Permeable Supports with 0.4 *μ*m pore size), and equal numbers of BMSCs were placed in the lower compartment. After coculture for 3 days, cells in the upper chamber were collected and incubated with CD34, CD38, CD135, and CD45RA antibodies (BD Pharmingen, San Diego, CA) to assess the percentage of common myeloid progenitor (CMP), Megakaryocyte-erythroid progenitor (MEP), and granulo-monocytic progenitor (GMP). In addition, 500 cells from the upper chamber after coculture were used for further CFU-C assay on H4435 methylcellulose. Fourteen days later, cells were gathered and stained with CD45, CD33, CD235a, and CD71 antibodies to detect erythroid lineage and myeloid lineage differentiation, after counting different hematopoietic colonies.

### 2.5. Apoptosis Assay

After coculture with CMML-BMSCs and HD-BMSCs in transwell plates for 3 days, cells in the upper chamber were gathered and stained with CD235a antibodies for 30 min at 4°C, then incubated with Annexin V-APC and 7-aminoactinomycin D (7AAD) for 15 min at room temperature. The apoptosis assay was performed on a FACS LSR II flow cytometer (Becton Dickinson, San Jose, USA).

### 2.6. Cytokine Array

Cytokine array was carried out to measure the differences of cytokine secretion between CMML-BMSCs and HD-BMSCs. In brief, BMSCs were cultured in an expansion medium in a 6-well plate at a density of 1.5 × 10^5^ cells/well. When cells reached 80% confluence, the expansion medium was replaced by a 2 mL conditional medium (CM, DMEM/F12 without FBS). CM derived from BMSCs was collected after 24 hr, and cytokines in CM were detected by Human Cytokine Array C1000 (RayBiotech, Norcross, GA, USA) according to the manufacturer's instructions. The signal was detected by ImageQuant LAS 4010 (General Electric Company, California, USA) and quantified with IQTL 7.0 software (General Electric Company). Different cytokine levels were further confirmed by enzyme-linked immunosorbent assay (ELISA, MultiSciences, HangZhou, China, or R&D Systems, Minneapolis, MN, USA).

### 2.7. Cytokine Rescue Assay

Additional cytokines were added back to the transwell coculture system, which was performed as described above. After coculture with interleukin-6 (IL-6), interleukin-8 (IL-8), and growth-regulated oncogene *β* (GRO-*β*) (100 ng/mL) for 3 days, CB CD34^+^ cells in the upper chamber were collected, and 500 cells were placed in H4435 methylcellulose for 14 days, followed by counting different hematopoietic colonies and flow cytometry analysis.

### 2.8. Statistical Analysis

Statistical evaluations were performed by Student's *t*-test for comparison between two groups and by ANOVA for comparison between multiple points. *p* values of less than 0.05 were considered significant.

## 3. Results

### 3.1. Culture and Phenotypic Analysis of BMSCs

**Figure 1:**
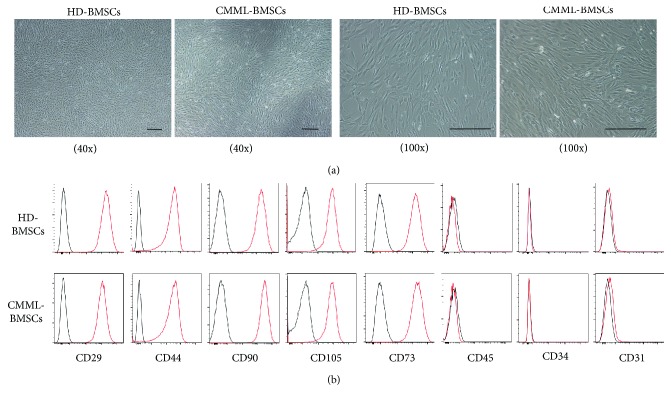
Characterization of CMML-BMSCs and HD-BMSCs. (a) Typical micrographs depicted the morphology of HD-BMSCs and CMML-BMSCs by light microscopy. Representative images obtained under 40x or 100x magnification are shown. Scale bar indicates 100 *μ*m. (b) Representative figures of cell surface marker analysis in CMML-BMSCs (lower panel) versus HD-BMSCs (upper panel) are shown. The red line represents the BMSCs stained with different antibodies while the black line represents the corresponding negative control stained with isotype-matched nonreactive fluorochrome-conjugated antibodies.

BMSCs from the BM of thirteen CMML patients and ten healthy donors were isolated and cultured *in vitro*. The clinical characteristics of CMML patients are listed in Supplemental Table 1. CMML-BMSCs exhibited similar spindle-shaped morphology as HD-BMSCs (Figure 1(a)). The expression pattern and mean fluorescence intensity (MFI) of cell surface markers were also similar between HD-BMSCs and CMML-BMSCs; for example, they were both positive for human BMSC markers including CD73, CD105, CD44, CD29, and CD90, whereas they were negative for endothelial cell surface marker (CD31), pan leukocyte marker (CD45), and HSPC marker (CD34) (Figure 1(b)).

### 3.2. CMML-BMSCs Displayed Impaired Hematopoietic Supportive Activity without Direct Cell-Cell Contact with CB CD34^+^ Cells

**Figure 2:**
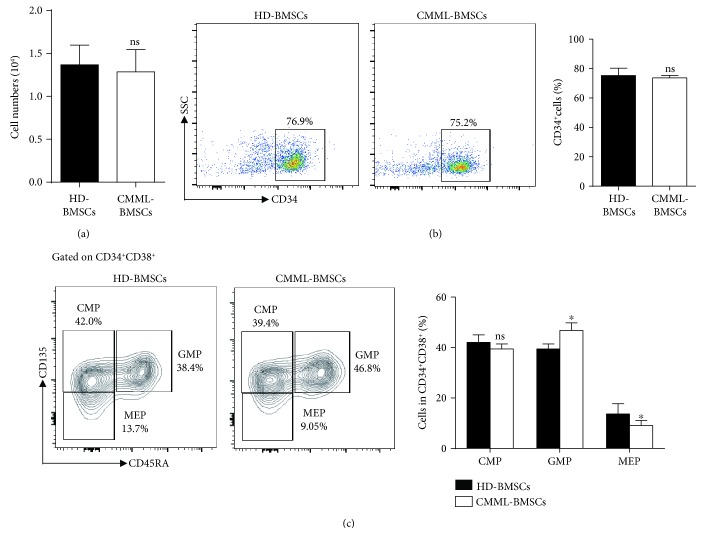
Analysis of cells in the upper chamber after a 3-day transwell coculture of CB CD34^+^ cells with HD-BMSCs or CMML-BMSCs. (a) Quantitation of cell numbers in the upper chamber after a 3-day coculture. (b) Flow cytometric analysis of CD34^+^ cell populations in the upper chamber after a 3-day coculture of CB CD34^+^ cells with HD-BMSCs or CMML-BMSCs. Quantification of the percentage of CD34^+^ cell population in the upper chamber is shown in the bar graph. (c) Flow cytometric analysis of CMP, GMP, and MEP populations in the upper chamber after a 3-day coculture of CB CD34^+^ cells with HD-BMSCs or CMML-BMSCs. Quantification of the percentage of CMP, GMP, and MEP populations in the upper chamber is shown in the bar graph (*n* = 5). Data are presented as the mean ± SEM, ^∗^ *p* < 0.05; ns: not significant.

Several mechanisms have been shown to be critical for the functions of the BM microenvironment, such as adhesion molecules, extracellular matrix, and soluble cytokines/chemokines. BMSCs are the major component of the BM microenvironment [4–8]. To determine whether soluble factors secreted by CMML-BMSCs have effects on their abnormal hematopoietic supportive activities, we performed noncontact transwell assay to separate CB CD34^+^ cells (upper chamber) from BMSCs (lower chamber). After 3 days of coculture with CMML-BMSCs or HD-BMSCs, the cells in the upper chambers were counted and analyzed by flow cytometry, then plated in methylcellulose for CFU-C assay. The total numbers of cells in the upper chamber of the transwell were identical, and the percentages of CD34^+^ cells (around 75% cells are CD34^+^ cells at this stage) were similar, after coculture of CB CD34^+^ cells with CMML-BMSCs or HD-BMSCs (Figures 2(a) and 2(b)). In addition, a significantly higher percentage of the GMP population was observed in the coculture of CB CD34^+^ cells with CMML-BMSCs compared to that with HD-BMSCs. In contrast, coculture of CB CD34^+^ cells with CMML-BMSCs induced a lower percentage of MEP population compared to that containing HD-BMSCs, whereas CMP populations were similar in both conditions (Figure 2(c)).

We then examined whether coculture with CMML-BMSCs affects the survival of early erythroblasts by flow cytometric analysis. The data showed that CMML-BMSCs did not result in a significant change in the apoptosis of the CD235a^+^ early erythroblasts as compared to that in the coculture with HD-BMSCs (Supplementary Figure 1).

**Figure 3:**
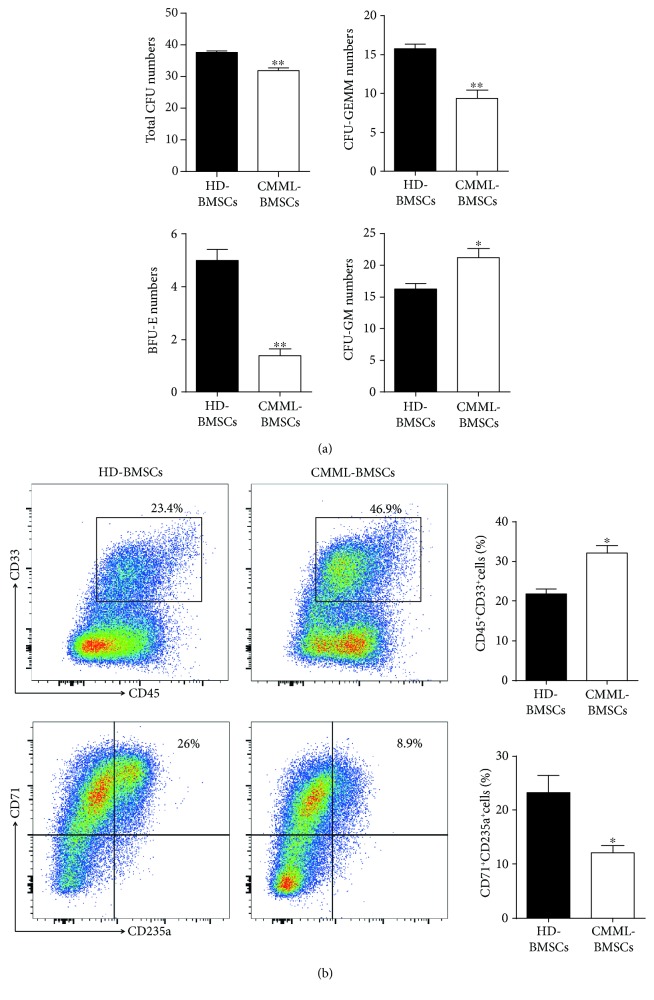
Analysis of hematopoietic colony-forming units on methylcellulose after coculture of CB CD34^+^ cells with HD-BMSCs or CMML-BMSCs. (a) After a 3-day coculture of CB CD34^+^ cells with HD-BMSCs or CMML-BMSCs, the CFU-C assays were performed with 500 cells collected from the upper chamber. After 14 days of culture in H4435 methylcellulose, colonies of CFU-GEMM, CFU-GM, and BFU-E derived from coculture with HD-BMSCs or CMML-BMSCs were enumerated. Data are presented as the mean ± SEM (*n* = 5), ^∗^ *p* < 0.05 and ^∗∗^ *p* < 0.01. (b) Representative flow dot plots show the percentage of myeloid (CD45^+^CD33^+^) or erythroid (CD71^+^CD235a^+^) cells after a 3-day coculture of CB CD34^+^ cells with HD-BMSCs or CMML-BMSCs followed by 14 days of culture on methylcellulose. Quantification of the percent myeloid and erythroid populations is shown. Data are presented as the mean ± SEM (*n* = 5), ^∗^ *p* < 0.05.

A Further 14-day culture on methylcellulose revealed that coculture of CB CD34^+^ cells with CMML-BMSCs led to significantly reduced numbers of total CFU-C, CFU-GEMM, and burst-forming unit erythrocyte (BFU-E) compared with HD-BMSCs (Figure 3(a), Supplementary Figure 2). However, a markedly increased number of colony-forming unit granulocyte and macrophage (CFU-GM) was observed after the coculture of CB CD34^+^ cells with CMML-BMSCs compared to those with HD-BMSCs (Figure 3(a), Supplementary Figure 2). In addition, flow cytometric analysis revealed that short-period coculture of CB CD34^+^ cells with CMML-BMSCs gave rise to more CD45^+^CD33^+^ myeloid cells (46.9%) than those cocultured with HD-BMSCs (23.4%) (Figure 3(b)). On the contrary, the percentage of CD235a^+^CD71^+^ cells (erythrocytes) derived from CB CD34^+^/CMML-BMSC coculture dropped to 8.9% (26% in HD-BMSC group) (Figure 3(b)). The results demonstrated that CMML-BMSCs exhibited an impaired hematopoietic supportive activity and preferentially promoted CB CD34^+^ cell differentiation toward the granulomonocytic lineage over erythrocytes, even without direct cell-cell contact.

### 3.3. Altered Cytokine Secretion in CMML-BMSCs

**Figure 4:**
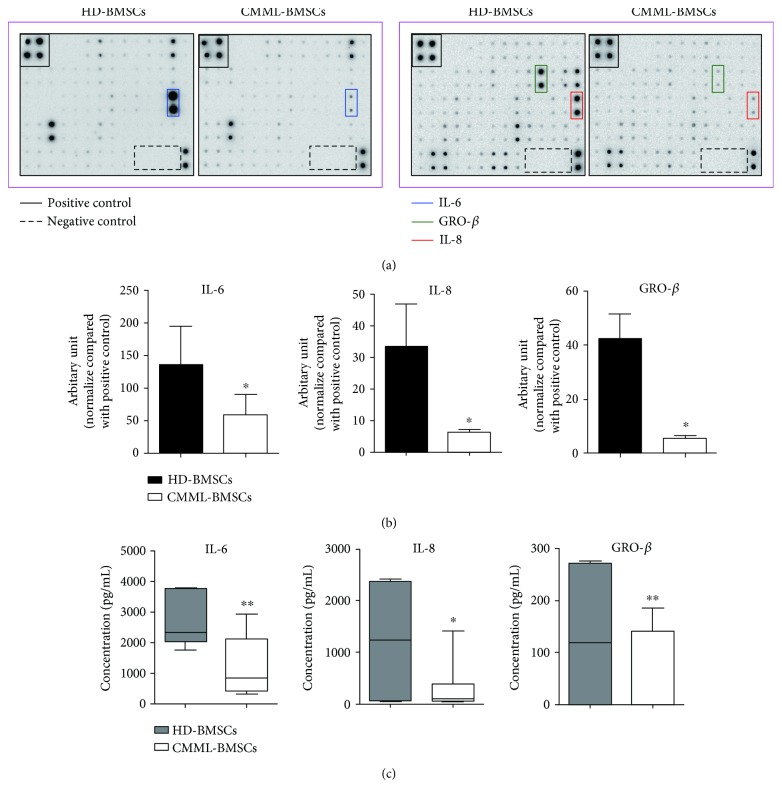
Comparison of cytokine secretion in CMML-BMSCs and HD-BMSCs. Cytokine array was performed to analyze the expression levels of soluble cytokines between HD-BMSCs and CMML-BMSCs. (a) Representative photographs showed that IL-6 (surrounded by blue lines), IL-8 (surrounded by red line), and GRO-*β* (surrounded by green line) significantly decreased in CMML-BMSCs compared with HD-BMSCs. (b) Quantitative analysis of the cytokine array using IQTL 7.0 software. Data are presented as the mean ± SEM (*n* = 5), ^∗^ *p* < 0.05. (c) ELISA assay was performed to validate the altered cytokine secretion in cytokine array (HD-BMSCs, *n* = 6; CMML-BMSCs, *n* = 7). Data are presented as the mean ± SEM, ^∗^ *p* < 0.05 and ^∗∗^ *p* < 0.01.

BMSCs can secret soluble cytokines to support HSPC self-renewal and differentiation. To identify the factor(s) that accounts for the impaired hematopoietic supportive activity of CMML-BMSCs, cytokine array was performed to compare the expression profile of soluble cytokines between HD-BMSCs and CMML-BMSCs. Three cytokines, IL-6, IL-8, and GRO-*β*, were decreased significantly in CMML-BMSCs compared with HD-BMSCs (Figures 4(a) and 4(b)). Further, ELISA assay confirmed that a 51% reduction of IL-6 and a 70% reduction of IL-8 levels were observed in CMML-BMSCs. Moreover, CMML-BMSCs produced only 50% GRO-*β* as compared with HD-BMSCs (Figure 4(c)).

### 3.4. Addition of IL-6, IL-8, or GRO-*β* Partially Restored the Impaired Hematopoietic Supportive Activity of CMML-BMSCs

**Figure 5:**
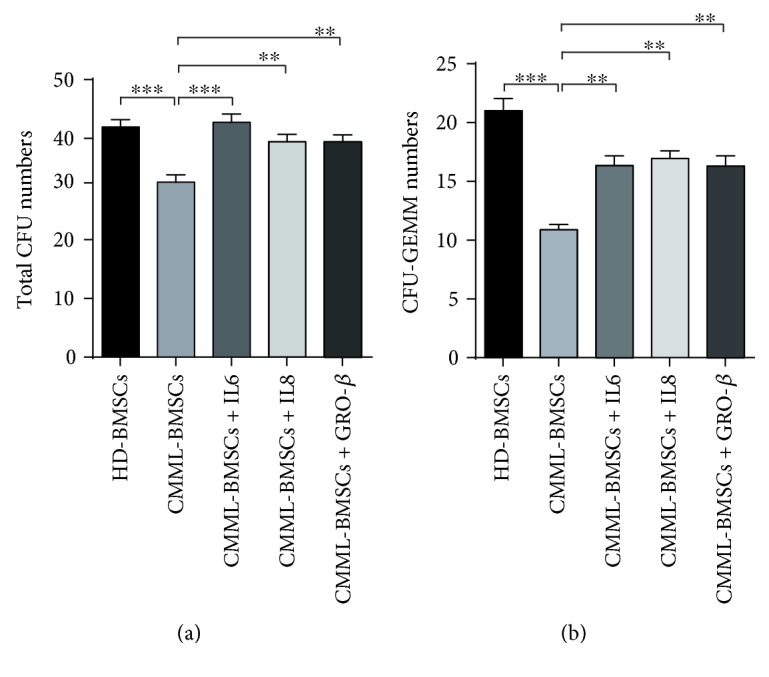
Rescue assay with additional cytokines. IL-6, IL-8, and GRO-*β* (100 ng/mL) were added into the transwell coculture medium, respectively. After 3 days, 500 cells in the upper chamber were collected for further CFU-C assay. Total CFU-C (a) and CFU-GEMM (b) derived from coculture with HD-BMSCs and CMML-BMSCs were enumerated. Data are presented as the mean ± SEM (*n* = 5), ^∗∗^ *p* < 0.01 and ^∗∗∗^ *p* < 0.001.

In order to verify the role of reduced cytokines in the impaired hematopoietic supportive activity of CMML-BMSCs, we performed noncontact coculture and CFU-C assay by supplementing the culture media of CMML-BMSCs with IL-6, IL-8, or GRO-*β*, respectively. As a result, addition of these cytokines significantly increased the numbers of total CFU-C and CFU-GEMM colonies (Figures 5(a) and 5(b)). However, there were no significant differences in the colony numbers of CFU-GM and BFU-E with or without the additional cytokines (data not shown). These results suggest that the deficient production of cytokines (IL-6, IL-8, and GRO-*β*) in CMML-BMSCs may be partially implicated in the pathological hematopoiesis in patients with CMML.

## 4. Discussion

The existence of BMSCs as one of the nonhematopoietic cells with hematopoietic supportive activity in the BM niche was first demonstrated by Till and McCulloch [21, 22]. As a vital component of the hematopoietic microenvironment, BMSCs are essential for HSPC maintenance, self-renewal, and differentiation. BMSCs have been used therapeutically to enhance hematopoietic reconstitution after HSC transplantation due to its immune modulating and hematopoietic supportive activity [23].

Though HSCs with intrinsic defects are the major origin for the initiation of myeloid malignancies [24–26], recent studies reported that the BMSCs derived from patients may also contribute to the pathogenesis of the diseases [12, 27, 28]. For example, molecular and functional deficits in BMSCs from MDS patients lead to impaired stromal support and may contribute to deficient hematopoiesis in MDS [29].

CMML is a clonal myeloid disorder with features of both MPN and MDS [18, 19]. However, the pathophysiology of CMML is still not well understood. We previously reported that CMML-BMSCs exhibited poor proliferative ability and hematopoietic supportive activity skewing myeloid differentiation [20], which is similar with studies that MDS-derived BMSCs and AML-derived BMSCs exhibited significantly defective growth characteristics compared to healthy donors [29, 30].

BMSCs can secrete multiple cytokines such as IL-6, IL-8, IL-11, IL-12, IL-14, IL-15, LIF, G-CSF, GM-CSF, M-SCF, FL, SCF, GRO, and osteoprotegerin to support HSC localization, self-renewal, and differentiation [31–33]. Lim et al. have shown that one of the cytokines, osteoprotegerin, could improve the hematopoietic supportive capacity of T-ALL BMSCs *in vivo* [34]. In order to verify whether the altered cytokine secretion of CMML-BMSCs was implicated in the impaired hematopoietic supportive activity of CMML-BMSCs, transwell coculture assay of CMML-BMSCs with CB CD34^+^ cells was carried out in the current study. Interestingly, noncontact coculture of CMML-BMSCs and CB CD34^+^ cells for only 3 days resulted in reduced CFU-GEMM numbers with a skewed differentiation toward the granulomonocytic lineage over the erythroid lineage, which is consistent with the granulomonocytic hyperplasia in patients with CMML.

Furthermore, we observed a significant reduction in the secretion of IL-6, IL-8, and GRO-*β* from CMML-BMSCs compared with HD-BMSCs. It has been reported that IL-6 and IL-8, secreted by BMSCs, can promote HSC stemness [35]. In particular, IL-6 induces hematopoietic cell proliferation, differentiation, and functional maturation [36]. IL-6 deficiency can also affect BMSC function and impair hematopoietic differentiation and supportive activity [37]. IL-8 can lead to HSC expansion in a CB-derived MSC-HSC coculture system *in vitro* [38]. In addition, IL-8 can also contribute to the mobilization of HSPCs *in vivo* [39]. GRO-*β* (also termed CXCL2), one of the key cytokine BMSCs secreted, is closely related with HSC homing, migration, and retention within the BM niche [40, 41]. In our study, adding these cytokines back to the noncontact transwell system partially restored the hematopoietic support of CMML-BMSCs.

In summary, our current study implies that the altered cytokine secretion of CMML-BMSCs may contribute to the pathogenesis of CMML, and correction of the microenvironment may serve as a potential therapeutic strategy for patients with CMML.

## Data Availability

All data supporting the findings of this study are available within the article and its Supplementary Information file or are available from the corresponding author upon request.

## Supplementary Materials

Supplementary Materials Supplementary Figure 1: apoptosis analysis (Annexin V/7AAD staining) on CD235a^+^ cells derived from CB CD34^+^ cells after a 3-day coculture with HD-BMSCs or CMML-BMSCs. Supplementary Figure 2: representative photographs of the hematopoietic colony-forming units derived from CB CD34^+^ cells cocultured with HD-BMSCs or CMML-BMSCs. Supplementary Table 1: BMSCs from the BM of thirteen CMML patients and ten healthy donors were isolated and cultured *in vitro*. Clinical information of CMML patients is listed in this table.
