# Supplementary material for: Alteration in the Cytokine Secretion of Bone Marrow Stromal Cells from Patients with Chronic Myelomonocytic Leukemia Contribute to Impaired Hematopoietic Supportive Activity
Source: Stem Cells Int. 2018 Jul 10;2018:5921392. doi: 10.1155/2018/5921392 (PMC6079359; doi:10.1155/2018/5921392)
Supplement: Supplementary Materials — Supplementary Figure 1: apoptosis analysis (Annexin V/7AAD staining) on CD235a+ cells derived from CB CD34+ cells after a 3-day coculture with HD-BMSCs or CMML-BMSCs. Supplementary Figure 2: representative photographs of the hematopoietic colony-forming units derived from CB CD34+ cells cocultured with HD-BMSCs or CMML-BMSCs. Supplementary Table 1: BMSCs from the BM of thirteen CMML patients and ten healthy donors were isolated and cultured in vitro. Clinical information of CMML patients is listed in this table. [file 5921392.f1.pdf]

Supplementary information

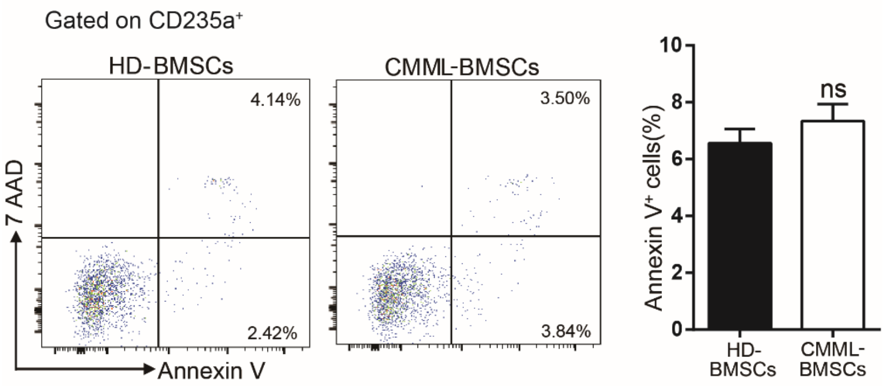

Figure S1.

Apoptosis analysis (Annexin V/7AAD staining) on CD235a+ cells derived from CB CD34+ cells after 3 days co-culture with HD-BMSCs or CMML-BMSCs. Data are presented as the mean ± SEM (n=5), ns, not significant.

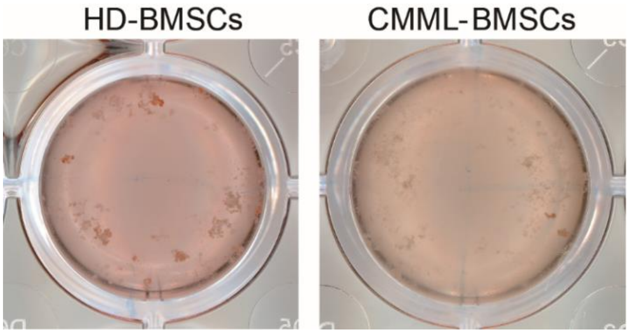

Figure S2.

Representative photographs of the colonies of cells after 3 days co-culture of CB CD34+ cells with HD-BMSCs or CMML-BMSCs followed by 14 days culture on methylcellulose.

Table S1. Clinical information of CMML patients

Patients	Gender	Diagnosis	Age at diagnosis	CPSS-P
M6	M	CMML	66	intermediate-2
M73	F	CMML	57	High
M97	M	CMML	62	intermediate-1
M144	M	CMML	72	intermediate-1
M260	M	CMML	78	High
M277	M	CMML	87	intermediate-1
M288	M	CMML	81	intermediate-2
M340	F	CMML	63	low
M347	M	CMML	29	intermediate-1
M368	M	CMML	61	intermediate-2
M429	F	CMML	57	intermediate-2
M432	M	CMML	42	intermediate-1
M479	M	CMML	75	intermediate-2

* Modified CMML-specific prognostic scoring system including platelet count (CPSS-P)
